# Case Report: Surgical Closure of Chronic Soft Tissue Defects Using Extracellular Matrix Graft Augmented Tissue Flaps

**DOI:** 10.3389/fsurg.2020.559450

**Published:** 2021-01-26

**Authors:** Micheal N. Desvigne, Krista Bauer, Kurt Holifield, Kari Day, Denise Gilmore, Ashley L. Wardman

**Affiliations:** ^1^Desvigne Plastic Surgery, Scottsdale, AZ, United States; ^2^Abrazo Arrowhead Hospital, Glendale, AZ, United States

**Keywords:** flap reconstruction, chronic wounds, soft tissue defects, extracellular matrix, ovine forestomach matrix

## Abstract

Chronic soft tissue defects are notoriously difficult to heal. Surgical reconstruction of chronic defects using tissue flaps is a routine approach for closure of challenging chronic defects. Due to the poor tissue quality of chronic defects and associated inflammation, infection and impaired blood supply the success of flap closure is marred by reported complication rates of 25–58%. Extracellular matrix (ECM)-based graft materials are commonly used for resolving chronic wounds and in plastic and reconstructive procedures to create a scaffold for tissue regeneration. We hypothesized combination use of ECM grafts with tissue flaps in a single-stage surgical procedure would reduce complications and improve outcomes in the closure of chronic soft tissue defects. We report a case series (*n* = 9) of chronic soft tissue defect reconstruction using this modified procedure of ECM graft augmented flap closure. Defects included pressure injuries and surgical dehiscence and ranged in wound age from 5 months to 7 years. Successful uncomplicated healing was achieved in six defects. Post-operative complications (dehiscence) occurred in two defects, however, these healed *via* secondary intention without additional surgical intervention. All healed defects exhibited acceptable cosmesis and “normal” function, with 100% patient satisfaction. Augmentation of tissue flaps with ECM graft materials in this modified single-stage procedure may improve outcomes and minimize typical complications encountered in flap closure of chronic defects attributed to inflammation, infection, hypoperfusion, and dead space.

## Introduction

Flap reconstruction is a well-established approach to the closure of chronic soft tissue defects however, post-operative complications such as infection, dehiscence, and re-occurrence are relatively common. The long-term success of flap closure is further complicated by patient co-morbidities such as obesity, diabetes and venous insufficiency. Retrospective analysis of 755 pressure injuries managed *via* flap closure demonstrated an overall complication rate of 25% at 30-day follow up (1). A prospective study of 276 pressure injuries closed by flap advancement demonstrated a complication rate of 58%, where wound dehiscence (31.2%) and re-occurrence (28.6%) were the most frequent complications (2). These complications associated with flap closure of chronic soft tissue defects are likely attributable to the poor quality of the underlying tissues which may be fibrotic and/or inflamed, the dead space potential between the advancing flap and underlying tissue, and poor vascularity of the tissues in general.

Extracellular matrix (ECM) grafts are absorbable bioscaffolds commonly used across a range of plastic and reconstructive procedures to scaffold soft tissue repair. These technologies provide a temporary scaffold for cellular infiltration and capillary formation while providing protective coverage and reinforcement of the defect until the bioscaffold is absorbed into the regenerating soft tissues (3). Many different ECM grafts are clinically available and differ in the origins of the source tissue (e.g., human, porcine, bovine, equine) and the processes used to decellularize the tissue to remove nuclear and cellular material while preserving the structure and composition of the tissue ECM.

Ovine forestomach matrix (OFM), is a decellularized ECM bioscaffold isolated from ovine forestomach tissue and has an established use in a range of clinical applications such as the management of acute and chronic wounds (4–9), skin grafting (10) and abdominal wall repair (11, 12). Previous studies have demonstrated OFM exerts a variety of biological functions. For example, OFM exhibits anti-inflammatory effects with broad-spectrum tissue protease modulation (13, 14), as well as stimulation of cell migration, differentiation and infiltration (15, 16). The matrix promotes neovascularization and is populated *via* cellular infiltration and completely remodeled into the regenerating tissues (16). Insights into the mechanisms behind these biological effects are provided by analysis of the structure and composition of OFM. To date, 151 different matrisomal proteins have been identified in the material that include a wide variety of collagens, adhesion proteins, and signaling molecules such as 12 growth factors including but not limited to fibroblast growth factor 2 (FGF2), platelet-derived growth factor (PDGF), epidermal growth factor (EGF), and connective tissue growth factor (CTGF) (15, 17). Structural studies have demonstrated that collagen fibril integrity and functional responses are preserved in OFM thus reflecting the retention of native ECM architecture (18). The material is highly porous and conducive to fluid imbibement and cellular infiltration, in addition to having robust mechanical properties suitable for incorporation into devices for load bearing indications (19).

Considering the regenerative properties of ECM grafts, it was hypothesized that complications following flap reconstruction of chronic wounds may be reduced by inclusion of an ECM graft to stabilize and augment the surgical flap and underlying tissues. This pilot case series presents our initial findings implementing this strategy.

## Materials and Methods

Retrospective data was collected from operation notes, clinical photography, and clinical records. A total of *n* = 9 defects from 9 patients were included in the case series (Table 1). All patients had various comorbidities known to complicate healing and were to undergo a planned flap reconstruction of a chronic defect. All defects were chronic and non-healing with an age range of 5 months to 7 years. All defects were prepared *via* sharp debridement or aggressive excision of chronic tissues. The OFM ECM graft (Myriad™ Soft Tissue Matrix, Aroa Biosurgery Limited, Auckland, New Zealand), was rehydrated in sterile saline (~ 5 min), trimmed to size, then placed into the base of the defect. Defects were then closed by local flap advancement and 3-0 Monocryl suture. Jackson-Pratt (JP) drains and incisional negative-pressure wound therapy (NPWT) were used as required (Table 1). Defects were monitored for up to 3–6 months (Table 1) for dehiscence, infection or recurrence.

**Table 1:** Participants.

**Participant (years)**	**Type**	**Comorbidities**	**Wound age**	**Location**	**Previous management**	**Surgical management**	**Time of last follow-up**
Male, 53(Case 1)	Surgical dehiscence post gastric band revision	Obesity	5 months	Abdomen	Alginate, surgical closure, cadexomer-iodine, saline gauze	Excision, ~8 × 2 cm defect, fasciocutaneous advancement flap, incisional NPWT, JP drain	Remained healed at 6 months
Female, 67	Surgical dehiscence post ileostomy	Diverticulitis, rheumatoid arthritis	48 months	Abdomen	NPWT	Excision, ~8 × 3 cm defect, fasciocutaneous advancement flap, incisional NPWT, JP drain	Remained healed at 6 months
Female, 70	Stage 4 pressure injury	Multiple sclerosis, paraplegia	6 months	Sacral and gluteal	Saline gauze	Excision, ~15 × 6 cm defect, fasciocutaneous rotation flap, incisional NPWT	Remained healed at 6 months
Female, 73(Case 2)	Stage 4 pressure injury	Parkinson's disease, Lupus, rheumatoid arthritis	7 years	Sacral	Previous flap reconstruction failed 1 month prior. Collagenase debridement, medical honey	Excision, ~8 × 4 cm defect, fasciocutaneous rotation flap, partial ostectomy, incisional NPWT	Postoperative dehiscence, Secondary healing at 3 months Remained healed at 6 months
Male, 62	Stage 4 pressure injury	Diabetes mellitus, paraplegia, Parkinson's disease, Lupus, rheumatoid arthritis	7 years	Sacral (×3)	Calcium alginate	Excision, ~8 × 4 cm, ~8 × 5 cm, and ~7 × 7 cm defects, fasciocutaneous advancement flaps, partial ostectomy, incisional NPWT	Remained healed at 6 months
Female, 26 (Case 3)	Surgical wound following open reduction internal fixation	Smoker	13 months	Ankle	Calcium alginate, ECM	Excision, ~8 × 8 cm defect, fasciocutaneous rotation flap, incisional NPWT	1 week—dehiscence with infection 11 weeks—wound healed Remained healed at 6 months
Female, 71	Surgical wound abdomen	Deep vein thrombosis, heart murmur, hypertension, diverticulitis, hyperthyroidism, obesity	5 months	Abdomen	Ca Alginate	Excision, ~6 × 2 cm defect, fasciocutaneous advancement flap, complex closure	Remained healed at 6 months
Male, 58	Sebaceous cyst, neck with active punctum	Obesity	4 years	Neck	Previous Incision and drainage	Excision, ~5 × 3 cm defect, fasciocutaneous advancement flap, complex closure	Remained healed at 3 months
Female, 64	Nodule excision	Previous breast cancer	12 months	Axilla	NA	Excision, ~9 × 4 cm defect, fasciocutaneous advancement flap, complex closure	Remained healed at 6 months

## Results and Case Examples

We hypothesized that the concurrent placement of an underlaid ECM graft during flap reconstruction of chronic wounds may reduce surgical complications by reducing inflammation of the proximal tissues and stabilizing the flap. Using this strategy, uncomplicated healing was achieved in *n* = 7 of the *n* = 9 study participants with defects (Table 1). Post-operative complication, namely dehiscence, occurred in *n* = 2 of the defects. However, both of these defects progressed to heal secondarily with no additional surgical intervention required. All healed defects demonstrated good cosmesis comparable to adjacent tissues. Assessment of healed defects demonstrated excellent functionality and motion and all patients were satisfied with their respective outcomes.

### Case 1

**Figure 1:**
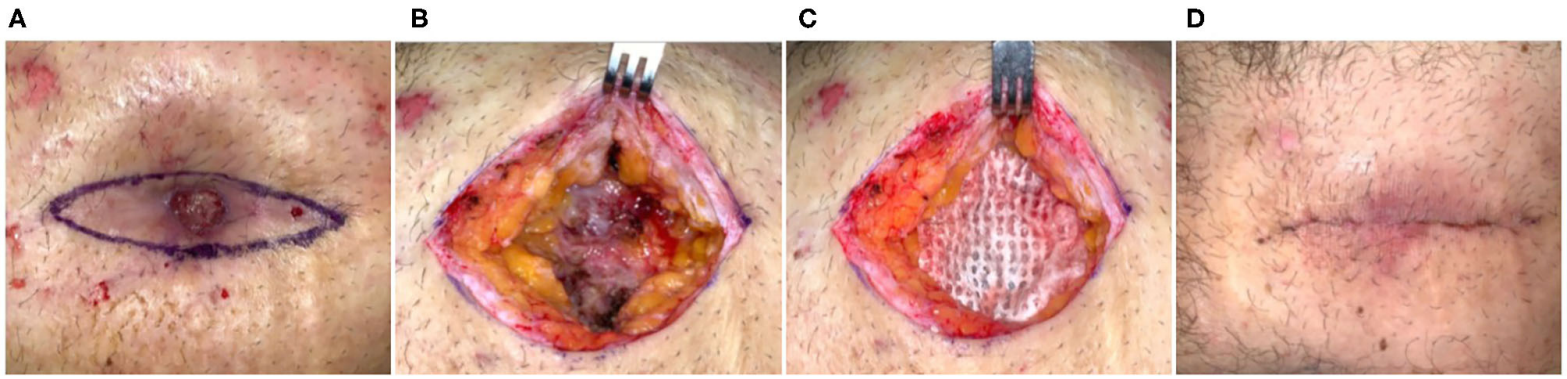
Flap advancement and ECM stabilization of an abdominal dehiscence. Case 1—**(A)** Erosion of the abdominal tissues resulting from a gastric band and failed previous surgical reconstruction of the ~11-month-old defect. **(B)** Wide excision of the defect down to the underlying fascia and adipose tissue. **(C)** Placement of the ECM graft into the base of the defect prior to flap advancement and closure. **(D)** Seven days post-op. Remained healed at 6 months.

Fifty-three-year-old obese male who had a single-port laparoscopic gastric band placement performed 10 years prior. The band eroded through the skin and was surgically removed but closure was complicated by secondary dehiscence. At the time of intervention, the patient had a non-healing abdominal wound for 11 months, a more recent revision surgery (5 months prior) had also failed with a dehisced surgical site. Previous management of the defect was carried out *via* alginate dressings, cadexmer iodine, and saline gauze. The defect was causing moderate pain, but was clean with no evidence of infection and hypertrophic granulation tissue was present (Figure 1A). Surgical incision was made at the defect margins, through subcutaneous adipose tissue and down to the fascia (Figure 1B). An ECM graft (Myriad™, “Thick”) was trimmed to fit the defect and placed onto the fascia (Figure 1C). Due to the depth of the defect and potential for dead space, second and third layers of ECM graft were prepared and placed in the defect in a layered arrangement. The defect was then closed by flap advancement with subcutaneous running suture with JP drain placed and incisional NPWT initiated. After seven days, cutaneous tissues demonstrated good apposition and tissues exhibited no inflammation (Figure 1D), the NPWT and drain were removed and patient was discharged with instruction to wear abdominal binder and 6 weeks heavy lifting/strenuous activity restriction. At 6 months, the site remained closed with no dehiscence or other complications.

### Case 2

**Figure 2:**
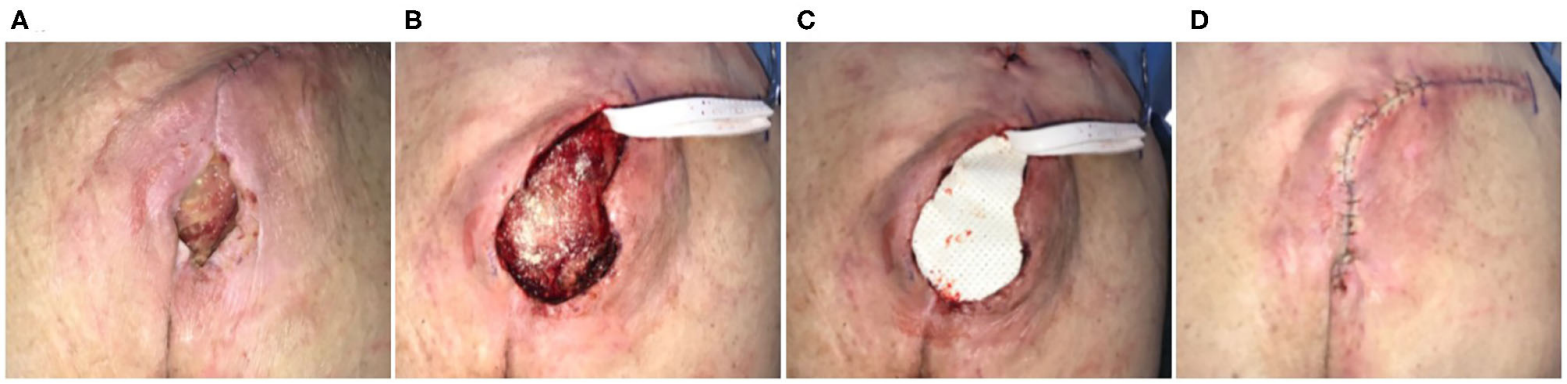
Surgical reconstruction of recalcitrant sacral pressure injury. Case 2—**(A)** Eleven-month-old pressure injury that had previously failed a flap reconstruction 1 month prior; secondary procedure performed using ECM graft to stabilize the flap. **(B)** Excision of ulcer with partial ostectomy. **(C)** Placement of the ECM graft and **(D)** flap closure.

Seventy-three-year-old female who was non-ambulatory secondary to Parkinson's disease, Lupus, and rheumatoid arthritis. Sacral pressure injury had been present for 7 years and had been previously managed with medical honey and enzymatic debridement. The patient had undergone a reconstructive procedure 1 month prior, using excision of the defect and flap coverage. The flap had subsequently dehisced and while there were no signs of infection, the defect was chronically inflamed (Figure 2A). Defect was excised with side margins down to the coccyx, with a partial ostectomy (Figure 2B). An ECM graft (Myriad™, “Thick”) was trimmed to fit the defect and into the base of the defect and covering the bone protrusion of the coccyx (Figure 2C). The excision was closed *via* a flap advancement with suture used to the close the primary incision line (Figure 2D). JP drains were placed and incisional NPWT initiated. A dehiscence of the site developed, which subsequently went onto heal *via* secondary intention at 3 months. At 6 months the defect remained healed.

### Case 3

**Figure 3:**
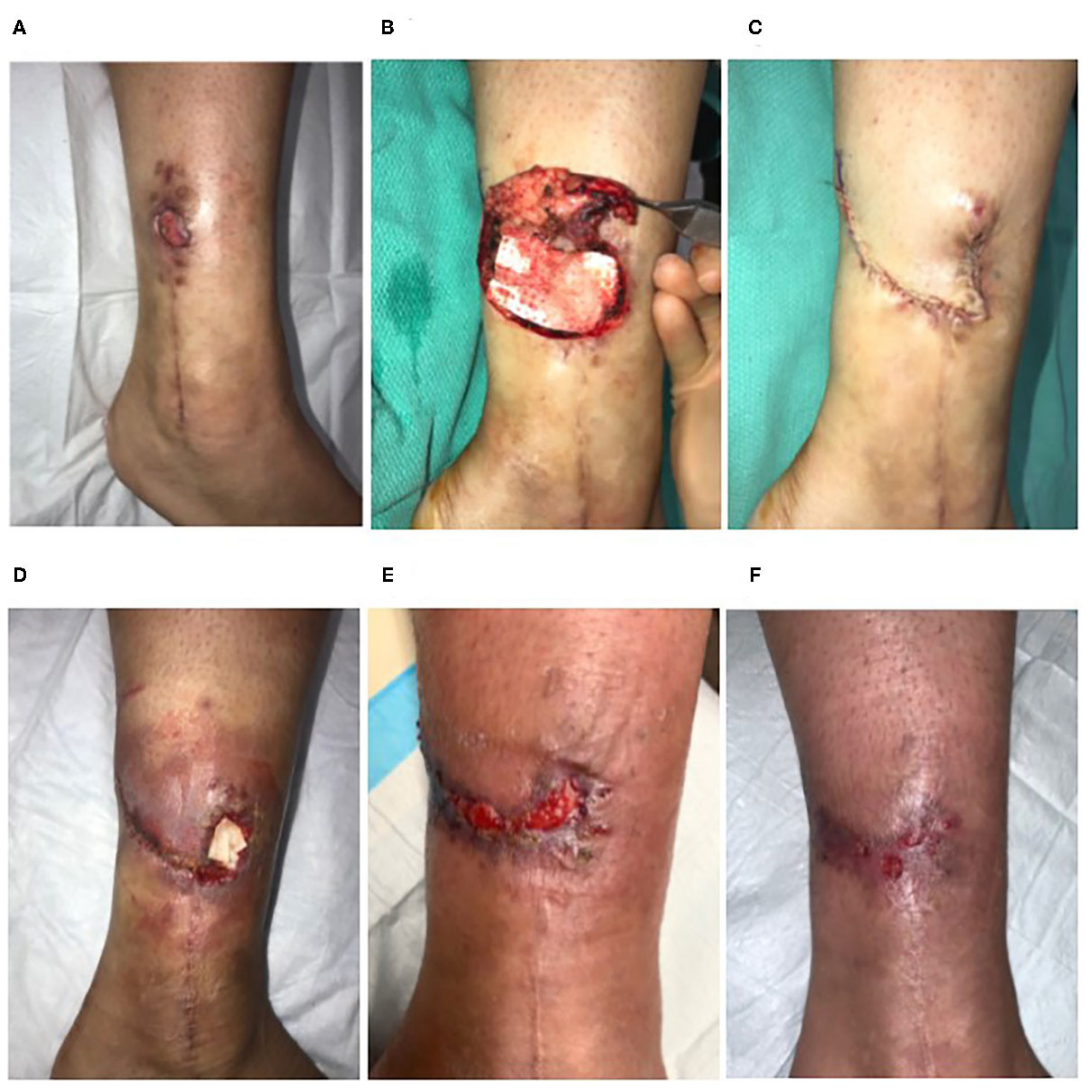
Reconstruction of a non-healing lower extremity surgical defect. Case 3—**(A)** Thirteen-month-old non-healing defect following open reduction and internal fixation surgery of the left ankle. **(B)** Surgical excision of proximal chronic tissues and placement of the ECM graft into the defect. **(C)** Flap advancement and closure. **(D)** The defect exhibited signs of infection and secondary dehiscence 1-week post-surgery and was treated with a second ECM graft. **(E)** At 6 weeks, the defect demonstrated marked improvement with healthy granulation tissue development. **(F)** Successful closure of the defect by secondary intention was achieved 8-weeks after the initial flap reconstruction without additional surgical intervention.

Twenty-six-year-old female with a 13-month-old non-healing full thickness defect following open reduction and internal fixation surgery of the left ankle (Figure 3A). Orthopedic hardware remained in place, and the wound was culture negative. The defect had previously been managed through use of alginate dressings. Surgical excision of proximal chronic tissues was carried out (Figure 3B). Trimmed ECM graft (Myriad™ “Thin”) was placed contacting the tissues of the defect bed and allowed to rehydrate *in situ via* absorption of blood components (Figure 3B). The defect was closed by flap advancement (Figure 3C) and underwent incisional NPWT for seven days. The defect exhibited signs of infection and secondary dehiscence 1-week post-surgery (Figure 3D). Additional ECM graft material was packed in the dehiscence cavity (Figure 3D) and covered with a gentian violet/methylene blue foam secondary dressing. Over the following weeks, the defect demonstrated marked improvement with healthy granulation tissue development (Figure 3E). Therefore, application of additional ECM graft material, hydrolyzed collagen dressings covered with a non-adherent dressing layer and continued use of gentian violet/methylene blue secondary dressings were used to heal the defect *via* secondary intention. Successful closure of the defect by secondary intention was achieved 8 weeks after the initial flap surgery (Figure 3F).

## Discussion

We hypothesized that complications following flap reconstruction of chronic defects may be reduced by the complementary use of an ECM graft to stabilize and augment the surgical flap and underlying tissues. This pilot case series explored using a one-stage procedure of flap reconstruction augmented with ECM graft placement. Simultaneous use of ECM grafts in flap closure is scarcely documented in the literature. The use of an amnion-derived ECM graft material has been shown to improve random flap survival in a preclinical murine model (20), however, this work primarily focused on combination use of amnion graft with mesenchymal stem cell supplementation and has not been translated into human studies. Various clinical reports exist of utilizing ECM grafts in the salvage of compromised or failed flaps (21, 22), but these reports describe reactive use of an ECM graft in a second intervention rather than a deliberate initial strategy. In periodontal surgery, ECM grafts are commonly used in combination with coronally positioned flaps for the treatment of gingival recession, acting to provide root coverage and increase the thickness of the gingiva (23, 24).

To our knowledge this is the first published work regarding the use of ECM grafts to augment flap reconstruction. The ECM graft utilized in the current study is derived from ovine (sheep) forestomach tissue, specifically the propria submucosa, a layer of ECM that extends through the forestomach tissue (15). Once isolated the propria submucosa undergoes a decellularization process to remove the ovine cells and nucleic acids leaving a layer of intact ECM. Decellularization utilizes a combination of detergents, chelating agents and salts to firstly lyse the ovine cells, then solubilize the cell membranes and nucleic acids (15). Grafts are fabricated using individual layers of ECM and presented either as a three- (~1.0 mm) or five-layer graft (~1.5 mm), in sizes up to 200 cm^2^. While advanced ECM bioscaffolds have typically been difficult to access due to cost, pricing of the OFM ECM graft (~USD$250–USD$2,500) enables novel usage of this technology in reconstructive procedures.

We hypothesized that an ECM graft may help to reduce flap complications *via* a combination of biological and physically mediated mechanisms. For example, the protease modulating effects of OFM (13) may rectify the underlying unbalanced environment in chronic soft tissue defects with high levels of tissue inflammation and proteolytic activity. Promotion of neovascularization by ECM material (16) may assist in establishment of blood supply from both the flap and the underlying tissues of the defect. Increasing the local blood supply to the defect through vascularization also minimizes risk of infection *via* increased perfusion of protective immune system elements and/or systemically administered antibiotics (25). Indeed, proper site preparation is vital for successful closure of chronic defects with positive post-debridement cultures being a proven predictor of failure of flap closure (26), as such our procedure included aggressive debridement and removal of chronic defective tissues. Additionally, placement of ECM graft material in the defect bed may provide occlusion of dead space between the flap and underlying soft tissues.

Results from the current pilot case series were encouraging, but the limited sample size must be noted. Additionally, five participants received incisional NPWT, the contribution of which cannot be fully assessed with this limited sample size. While incisional NPWT is known to reduce surgical site infections, the risk of dehiscence of surgical primary closures has been shown to be equivalent to standard wound dressings (27).

## Concluding Remarks

Flap reconstruction is an effective and prevalent method for the repair of chronic soft tissue defects. The present case series piloted use of an ECM graft to augment flap reconstruction of chronic soft tissue defects. Outcomes of these initial cases may warrant future controlled studies for evaluation of this technique relative to unmodified flap closure.

## Data Availability Statement

The original contributions presented in the study are included in the article/Supplementary Materials, further inquiries can be directed to the corresponding author/s.

## Ethics Statement

All patients provided written informed consent for their images and data to be used for research and publication purposes.

## Author Contributions

MD contributed to the design of the study, clinical management, data collection, data analysis, and preparation of the manuscript. KB and AW contributed to case management and data collection. KH, KD, and DG contributed to clinical management. All authors contributed to manuscript revision, read, and approved the submitted version.

## Conflict of Interest

MD consults for Aroa Biosurgery Limited (Auckland, New Zealand). Myriad™ Soft Tissue Matrix was provided by Aroa Biosurgery Limited. The remaining authors declare that the research was conducted in the absence of any commercial or financial relationships that could be construed as a potential conflict of interest.
